# Adaptive occupational alignment: a processual model of workforce reintegration after spinal cord injury

**DOI:** 10.3389/fpubh.2026.1831601

**Published:** 2026-07-16

**Authors:** Armand Bam, Ella van der Merwe

**Affiliations:** Stellenbosch University, Stellenbosch Business School, Cape Town, South Africa

**Keywords:** disability inclusion, employment, self-management, spinal cord injury, workforce reintegration

## Abstract

**Background:**

Despite progressive policy commitments, employment outcomes for individuals with spinal cord injuries (SCI) remain low, highlighting the need to examine both systemic and individual-level factors influencing return to work. This study explores how workforce participation is sustained through the interaction of structural conditions, relational supports, and adaptive agency among individuals with spinal cord injury following rehabilitation.

**Methods:**

A qualitative multiple-case design was employed. In-depth interviews were conducted with 10 individuals who had returned to work following spinal cord injury. Data were analysed thematically.

**Results:**

Three overarching themes were identified: Structural Conditions and Inclusive Design, Social Support and Relational Resilience, and Agency, Adaptation, and Career Growth. The findings demonstrate that workforce sustainability emerges through the interaction of accessibility, relational support, and individual agency, yet remains vulnerable to persistent structural and symbolic barriers.

**Conclusion:**

Drawing on these findings, the study advances an Adaptive Occupational Alignment model, conceptualizing workforce reintegration as an ongoing, multi-level process through which health needs, workplace practices, and relational supports are dynamically aligned to sustain participation over time.

## 1 Introduction

Workforce reintegration after spinal cord injury (SCI) remains a significant challenge despite advances in rehabilitation (1). While a growing body of literature has examined vocational rehabilitation and return-to-work interventions following SCI (2–4), less attention has been given to how individuals experience workforce participation as an ongoing occupational, relational, and health-related adjustment process after formal rehabilitation (1, 5, 6). In contexts marked by structural inequality and limited workplace accommodation, such as South Africa, this gap is particularly pronounced as reintegration unfolds within complex institutional and social constraints with direct implications for long-term health, wellbeing, and sustained workforce participation.

Despite progressive legislation, employment outcomes for persons with disabilities remain poor globally (7–9). Although policy frameworks increasingly endorse workplace inclusion, their implementation is often fragmented and uneven (68). As a result, many organizations continue to struggle with addressing accessibility requirements, challenging disability-related stigma, and removing structural barriers. This disconnects between policy aspirations and organizational realities contributes to exclusion, employment instability, and preventable health strain among persons with disabilities (42).

Employment, when accessible, contributes significantly to physical and mental wellbeing, self-worth, and broader socioeconomic participation (10, 11). Nevertheless, people with SCI continue to experience disproportionately high levels of unemployment and underemployment, even after completing formal rehabilitation (3, 9). Evidence from Switzerland suggests that targeted workplace interventions can improve labor market participation by addressing environmental constraints (12). However, similar challenges persist in South Africa despite a supportive legislative framework (13). This disparity highlights that successful workforce reintegration depends not only on individual functional capacity but also on the sustained alignment of workplace conditions, support systems, and evolving health needs.

Building on Hilberink and Cardol's (14) critique of the individualization of agency and extending Trezzini et al.'s (12) analysis of environmental barriers, this study shifts attention from isolated barriers and facilitators toward examining how workforce participation is continually negotiated through the interaction of structural conditions, relational supports, and adaptive agency. In doing so the study examines how agency is enacted, shared, and constrained within workplace contexts, and how these dynamics shape the sustainability of work participation after SCI.

Situated within the South African context, the study contributes empirical insight from the Global South by illustrating that legal and environmental supports, while necessary, are insufficient in the absence of organizational and relational mechanisms that enable meaningful participation.

### 1.1 Conceptualizing workforce reintegration post SCI

In examining SCI management and its broader consequences, this review engages with three interlinked domains that shape how health, work, and participation are negotiated following SCI. First, it outlines the foundations of SCI management and recovery, tracing the continuum from acute care to rehabilitation and self-management. Second, it reviews scholarship on psychosocial dimensions and vocational reintegration after SCI. Third, it considers workplace inclusion and broader structural contexts, including labor market and policy conditions. Together, these areas provide the framework for reviewing existing knowledge on workforce reintegration following SCI.

#### 1.1.1 Foundations of SCI management and recovery

SCI management increasingly emphasizes a continuum of care that includes acute medical treatment, rehabilitation, and long-term self-management strategies. Given the largely irreversible nature of SCI, early diagnosis and intervention are critical (15). In the context of SCI management, medical efforts are directed toward stabilization and minimizing further damage during the acute phase of injury. This approach reflects that the severity of impairment correlates with the extent and type of injury (16). However, the implications of SCI extend far beyond the initial physiological event itself (17). Delayed or inadequate diagnosis can amplify the disruption to a person's social and economic life, particularly in relation to long-term workforce participation, economic security, and social inclusion.

Advances in surgical and rehabilitative techniques have improved survival rates, but the transition from hospital to home remains complex (18, 19). While acute-phase interventions aim at spinal stabilization (20), post-discharge care demands a more sustained engagement with self-management as individuals begin to navigate everyday life beyond clinical settings. Defined as an individual's ability to manage symptoms, treatments, and broader health-related challenges, self-management has become central to improving quality of life and functional independence, and participation in everyday activities, including work (21). However, many available rehabilitation programmes remain generic and do not sufficiently address the unique demands of SCI across diverse social and employment contexts, or the social and occupational reintegration process (22).

A shift toward patient-centred rehabilitative care has reshaped traditional approaches to SCI recovery. Rehabilitation is now recognized, not only as a physical recovery process, but as a relational and identity-forming experience with implications that extend beyond clinical recovery. Patient-centred models emphasize aligning rehabilitative goals with the individual's own aspirations, cultural background, and socio-economic context, including future work participation and role re-engagement (23). Engaging patients in co-designing their treatment increases long-term adherence and supports the development of self-efficacy essential for navigating complex post-injury health, social, and occupational realities (24, 25).

#### 1.1.2 Psychosocial dimensions and vocational reintegration

Emotional and psychosocial adjustment is a critical but often overlooked component of SCI rehabilitation. Individuals with SCI frequently face mental health challenges including anxiety, depression, and identity loss, which can disrupt community participation, occupational functioning, and sustained employment (26, 27). In response, multidisciplinary rehabilitation must incorporate mental health professionals who provide trauma-informed counseling and support the development of coping strategies as individuals prepare to re-enter or remain in the workplace (28).

Vocational outcomes are also shaped by the work of social workers, who assist with navigating systems, relationships, and community reintegration (29). Despite recognition of employment as central to recovery, vocational rehabilitation tailored to SCI remains limited (2). This gap constrains the coordination between rehabilitation, work readiness, and labor market participation, limiting pathways to economic independence.

#### 1.1.3 Workplace inclusion and structural challenges

SCI often precipitates a sharp rupture in an individual's professional life. While some biomedical variables such as injury level and pre-injury employment shape the likelihood of returning to work, they do not fully explain labor market exclusion, particularly when workplace structures and jobs remain inflexible (30, 31). Global employment rates among individuals with SCI remain low, with only 38% of working aged individuals employed, in contrast to over 56% in the general population (9, 32). In the context of South Africa, this divide is further compounded by inequities in access to rehabilitation, education, and employment pathways (33). These disparities point to structural constraints within labor markets that extend beyond individual impairment and continue to shape unequal access to work.

Return to work is rarely linear and depends on the coordination of medical recovery, psychosocial readiness, and employer support (34, 69). Health status, mental health challenges, and discriminatory workplace practices also intersect to shape labor market experiences over time (6). The persistence of these challenges highlights the need for adaptive workplace practices and labor market policies that can respond to changing health, capacity, and participation needs.

Self-management strategies such as decision-making, goal setting, and resilience can further support long-term employability, especially when reinforced by accessible workplaces and peer support networks over the course of an individual's working life (35, 72). While self-efficacy is necessary, it is insufficient in the absence of enabling structural conditions (36). A coordinated ecosystem of healthcare professionals, vocational counselors, employers, and policymakers is required to build inclusive employment pathways that support ongoing regulation of health, work demands, and participation (37, 38, 67).

For individuals with SCI who do return to work, workplace accommodations are a decisive factor in job retention and satisfaction over time. These can range from ergonomic modifications to flexible scheduling and remote work options (39, 40). Studies consistently show that inclusive environments support productivity, reduce health outcomes, and increase workplace satisfaction (41). Yet accommodations are inconsistently implemented, and their availability often depends on employer discretion rather than institutionalized rights or standard organizational practices (42). Beyond access to accommodations, employment outcomes are also shaped by underemployment and insecure work conditions (66). Individuals with SCI may secure employment but face limited advancement, unstable contracts, or roles that underutilize their skills and qualifications (33, 43).

The proliferation of assistive technologies has enabled greater autonomy for people with SCI. Technology has become a powerful enabler of both health and vocational reintegration by supporting the coordination of care, work tasks, and daily routines, from mobile health apps that assist with medication management to platforms offering job readiness tools and access to online support (44, 45). Wearable devices and communication technologies support not only health management but also remote work and flexible participation in professional spaces. However, the promise of digital inclusion remains unevenly realized. Structural inequalities such as inaccessible design, limited digital literacy, or lack of reliable internet access compound existing barriers to work and care (46). Without a critical lens on digital equity, the expansion of assistive technologies risks reproducing existing patterns of exclusion within work and care systems.

A formal commitment to disability inclusion is reflected in the SA policy landscape of this study. Frameworks like the Employment Equity Act (1998) and its associated Code of Good Practice which mandate reasonable accommodation and employment equity planning (47). However, the translation of these policy commitments into workplace practice is hindered by poor enforcement, limited employer awareness, and a reliance on voluntary compliance (5) (Author). As a result, barriers remain deeply embedded in institutional cultures, revealing a persistent gap between legislative intent and structural transformation, particularly in relation to everyday work practices and decision-making (13).

### 1.2 Theoretical perspectives: framing self-management within social and structural contexts

This study draws on three interrelated theoretical perspectives: the social model of disability, self-efficacy theory, and resilience theory to explore workforce reintegration as an ongoing occupational process for individuals with spinal cord injury. Together, these perspectives provide a multi-layered lens for examining how structural conditions, personal agency, and adaptive capacity shape the sustainability of post-rehabilitation employment.

At its foundation, the social model of disability (48) challenges individualized or deficit-based views of disability by framing it as the outcome of systemic exclusion, environmental inaccessibility, and discriminatory institutional structures. In this model, disability is not inherent to the body but is instead produced by social arrangements that fail to accommodate diversity within everyday social and organizational contexts (49). For individuals with SCI, barriers to employment often stem not from the injury itself, but from inaccessible infrastructure, a lack of reasonable accommodations, and marginalizing workplace cultures that fail to align job design with diverse functional capacities. Within this study, the social model provides a foundation for understanding workforce reintegration as a structurally mediated process, highlighting how organizational practices and labor market arrangements shape opportunities for participation.

Self-efficacy theory complements this structural lens by offering insight into how personal beliefs influence behavior, especially in the face of challenge (50). Self-efficacy refers to an individual's confidence in their ability to achieve goals and manage tasks within specific social and occupational contexts. For people with SCI, higher levels of self-efficacy are associated with better self-management, greater adaptability to changing work routines, and more sustained engagement with employment under conditions of change and uncertainty. Self-efficacy also shapes how individuals respond to barriers, with those who report greater self-belief more likely to engage in problem-solving and persist despite setbacks (51). In this way, self-efficacy functions not only as a psychological resource but as a mediator of social and occupational reintegration.

Resilience theory further enriches this analysis by focusing on the dynamic processes through which individuals adapt positively to adversity (52). Rather than a fixed trait, resilience is understood as the capacity to recover from challenges and maintain purpose and direction in the face of uncertainty across changing life and work circumstances. For individuals with SCI, resilience is central to navigating the demands of both rehabilitation and workforce participation. Resilience is fostered through access to resources, supportive social networks, and coping strategies that enable individuals to recover from setbacks and maintain engagement with work. In professional contexts, this adaptive capacity supports constructive responses to discrimination, fluctuating health, and systemic inflexibilities.

Together, these three theoretical perspectives frame self-management not as an isolated trait, but as a socially situated and structurally mediated process that unfolds across health, work and social domains. Together, they inform this study's research objectives by foregrounding how individual agency, systemic exclusion, and adaptive capacity interact in shaping sustainable workforce participation. By integrating these lenses, the study conceptualizes autonomy, resilience, and self-efficacy as relational and context dependent mechanisms within a process of adaptive occupational regulation, rather than as individual attributes alone.

## 2 Methodology

The study was underpinned by an interpretivist qualitative orientation, drawing on a multiple case study design to explore how workforce participation is sustained through the interaction of structural, relational, and agentic factors following spinal cord injury. This design was selected to enable analytic generalization across varied reintegration trajectories (53, 54). The design was interpretivist in orientation, seeking to understand reintegration as a lived, relational, and structurally mediated process (55).

In this study, each participant constituted a bounded case, defined as an individual with SCI who had completed formal rehabilitation and returned to paid employment within the South African labor context. The case boundary was delimited by the participant's post-rehabilitation employment trajectory, enabling examination of how health management, workplace practices, and relational supports interacted within everyday occupational settings. A multiple case design was selected to enable analytic generalization across varied reintegration trajectories (53, 64). Including 10 cases supported both literal replication (similar reintegration experiences under comparable conditions) and theoretical replication (contrasting trajectories under differing structural or relational conditions), facilitating identification of patterned similarities and meaningful divergence across cases. This design underpinned the development of the Adaptive Occupational Alignment model (63).

Given the study's focus on lived experience and meaning-making, in-depth interviews constituted the primary and appropriate data source. The design prioritized experiential depth within bounded cases rather than documentary corroboration, consistent with interpretivist case study methodology where the phenomenon under investigation concerns subjective and relational processes (74).

### 2.1 Sampling and participants

A snowball sampling strategy was employed, initiated through author two (EvM) professional networks. Participants were invited by email or telephone. This approach enabled access to participants with specific experiential knowledge within a population that can be difficult to reach through probability-based sampling (56).

Participants were eligible if they:

- Had been diagnosed with SCI (traumatic or non-traumatic),
- Completed rehabilitation at a healthcare facility in South Africa,
- Were employed in paid work post-rehabilitation,
- Were aged 18 years or older.

Variation was sought across injury severity, employment sector, and socio-demographic background to strengthen theoretical replication. The final sample comprised 10 participants (3 female, 7 male), aged between 21 and 60 years, with the majority between 31 and 50 years. Injury levels were distributed across cervical (*n* = 5) and thoracic (*n* = 5) categories. Participants were employed across small and medium enterprises and corporate organizations. Living arrangements varied, including participants who lived alone, with partners or spouses, and with extended family members, enabling exploration of diverse relational contexts. Participant characteristics are presented in Table 1.

**Table 1:** Demographic and employment characteristics of participants.

Participant ID	Age range	Sex	Self-identified race	Neurological level	Classification	Years post injury	Employment sector	Work arrangement (Remote/In-Person)
1	21–30	Female	White	T12	Paraplegia	2	Private sector (Energy, oil and gas)	In-Person
2	41–50	Male	White	C3/4	Tetraplegia	28	Small and medium enterprise (Social media)	Remote
3	51–60	Female	Colored	C6	Tetraplegia	36	Private sector (Banking)	Hybrid
4	31–40	Male	Black	T4/5	Paraplegia	14	Small and medium enterprise (Information and Communication Technology)	In-Person
5	21–30	Male	Colored	C7	Tetraplegia	1	Government	In-Person
6	51–60	Male	White	C5/6	Tetraplegia	40	Private sector (Higher education)	In-Person
7	31–40	Female	Colored	T7	Paraplegia	11	Small and medium enterprise (Administrative)	In-Person
8	51–60	Male	White	T12	Paraplegia	18	Small and medium enterprise (Wheelchair business)	In-Person
9	41–50	Male	White	T12	Paraplegia	26	Private sector (Fast Moving Consumer Goods)	In-Person
10	31–40	Male	Colored	C7/8	Tetraplegia	15	Government	In-Person

Sex and race/ethnicity data were self-reported by participants. Race as a category reflects terminology used in the South African census and employment equity context.

### 2.2 Data collection

Data were generated through semi-structured, in-depth interviews guided by a literature-informed protocol (57). The interview schedule explored experiences of rehabilitation, self-management, workforce reintegration, workplace accommodation, and structural challenges.

Interviews were conducted by researcher two (EvM) either in person or via secure online Teams/ Zoom platforms and lasted between 60 and 90 min. Interviews were audio-recorded on Mp3 device where in person interviews occurred with informed consent. Interviews were transcribed and returned to participants for review. The semi-structured format allowed consistency across cases while enabling participants to elaborate on issues salient to their individual reintegration trajectories. The interview guide is presented in Appendix A. Field notes were taken capturing behaviors and environmental context.

### 2.3 Reflexivity and researcher positioning

Reflexivity was intentionally integrated throughout the research process due to the sensitive and relational nature of the topic (65). In this study, the first author (AB - male) specializes in the qualitative research of the inclusion of people living with disability and chronic illness, advocating for workplace equity and centring participant voices (73). The second author (EvM - female) is a medical practitioner with experience working in public and private settings with spinal cord injuries and rehabilitation programmes. EvM's positioning provided contextual insight into participants' accounts but also introduced potential power asymmetries and interpretive bias, which were managed through scheduled debriefing sessions with the first author (AB). These debriefing sessions interrogated assumptions, examined alternative explanations, and strengthened analytic rigor and transparency. Moreover, the author (AB) ensured ongoing methodological oversight and facilitated critical dialogue regarding emerging interpretations in the data. As a further measure, both authors participated reflexive journaling throughout data collection and analysis.

### 2.4 Data analysis

Data analysis followed a two-stage process consistent with multiple case study methodology (70). During stage one referred to as within-case analysis, each case was first analysed individually to construct a coherent narrative of the participant's reintegration trajectory. This stage involved examining how structural conditions, relational supports, and adaptive strategies interacted within that specific case context. Within-case analysis enabled depth and preserved contextual integrity before cross-case synthesis.

The second stage, cross-case analysis followed the within-case analysis stage, where cross-case comparison was conducted to identify convergent and divergent patterns. Interview transcripts were analysed using Braun and Clarke's (58) six-phase thematic approach, supported by ATLAS.ti for systematic data management. The first phase involved data familiarization through repeated transcript reading. During initial coding, salient features related to health management, workplace navigation, relational dynamics, and adaptive strategies were identified. Codes were then clustered into higher-order categories and compared across cases. Matrices were then developed to facilitate structured comparison across structural, relational, and intrapersonal dimensions. This cross-case analysis enabled analytic generalization, allowing the identification of recurring alignment processes that informed the development of the Adaptive Occupational Alignment model.

Consistent with (71), the study sought not only to identify recurring themes but to develop a richly textured understanding of how workforce participation following SCI is negotiated through structural, relational, and agentic processes. Themes were refined collaboratively by both researchers to ensure coherence, internal consistency, and theoretical sensitivity.

Reporting followed the Consolidated Criteria for Reporting Qualitative Research (COREQ) guidelines (59) (see Supplementary material 1).

### 2.5 Ethical considerations

Ethical approval was obtained from the University's Research Ethics Committee: Social, Behavioural and Education Research (REC: SBER 31536). Participants received detailed information sheets outlining the purpose and scope of the study, and written informed consent was obtained prior to participation. As ethical practice extended beyond procedural compliance, attention to relational ethics, participant comfort, and respectful representation of lived experience was included. Care was taken to ensure anonymity and to avoid deficit framing in reporting participants' accounts.

## 3 Findings

Findings from 10 in-depth interviews revealed that workforce reintegration following SCI was sustained through the continual interaction and recalibration of structural conditions, relational supports, and adaptive agency. Rather than operating as isolated factors, these domains dynamically shaped participants' capacity to maintain workforce participation over time. Three interrelated themes were identified (Table 2).

**Table 2:** Themes shaping workforce reintegration after spinal cord injury.

Theme	Category	Description
Structural conditions and inclusive design	Physical accessibility and design flaws	Participants encountered poorly designed infrastructure, inaccessible transport, and inadequate assistive devices that constrained daily work participation.
	Policy vs. practice gaps	Legal frameworks existed, but inconsistent implementation and organizational discretion produced systemic barriers.
Social support and relational resilience	Support networks	Early reliance on peer support evolved into sustained family and collegial involvement that supported continued workforce participation.
	Managerial empathy and relational understanding	Supportive managers and colleagues shaped inclusion through empathy, flexibility, and informal accommodation practices.
Agency, adaptation, and career growth	Self-efficacy and adaptation	Participants described adaptive mindsets, strategic planning, and growing confidence in managing health and work demands.
	Workplace education and culture shift	Awareness initiatives and informal education improved team dynamics, enabling greater agency, and participation.

### 3.1 Structural conditions and inclusive design

Participants described structural conditions not as static background factors, but as dynamic determinants of whether workforce participation could be sustained over time. Accessibility, transport, flexibility, assistive devices, and workplace policy continuously interacted with health fluctuations and workplace expectations, requiring ongoing adaptation and recalibration.

#### 3.1.1 Accessibility

Accessibility was a recurring concern in participants' accounts of self-management, and workforce integration. P1 described the uncertainty of navigating work environments stating, “*it can be tricky for me to get to where I need to go a lot of the time because I'm not sure if I'll have access.”* Even when accessible areas were provided, their placement sometimes produced feelings professional isolation. As P1 noted:

“*I feel a little bit like I've been pushed off to the side”*

Employer-led adaptations were described as important in reducing daily uncertainty and strain. P9, who had been with their company for 21 years, explained:

“*My work has been very open to adapting in terms of certain needs, and for the most part, it's built around accessibility.”*

Participants also emphasized that accessibility did not always require extensive structural redesign. As P4 observed, relatively modest adjustments, such as accessible parking or bathrooms, could enable continued participation.

#### 3.1.2 Transport

Transport emerged as a significant barrier, although its impact differed according to the level of injury and access to financial resources. Participants with lower level lesions, who were able to drive adapted vehicles, described transport as manageable, with accessibility concerns centred primarily on parking. As P8 explained:

*And I think there's a misconception with…disabled parking. Everybody thinks it has to be right in front because it makes it easy. That's not the case… it's a case of just having that freedom [space] to be able to move*.

For participants with higher-level lesions or without the resources to modify vehicles, transport posed more persistent constraints. Many relied on family support or public transport systems that were not consistently accessible. As P2 noted, “*Transport to work is a big issue.”* For others, transport directly shaped the feasibility of continued employment. P3 reflected:

*If I have to come into work every day and remote work is not gonna be an option anymore. I don't know what I'm gonna do. I'll probably have to resign because there is no way I can go into the office every day*.

Across cases, access to private or reliable transport influenced whether employment remained sustainable or uncertain.

#### 3.1.3 Workplace flexibility

Workplace flexibility emerged as a key facilitator of workforce participation, particularly where rigid schedules or fixed office environments intensified health and mobility constraints. Work-from-home arrangements were especially significant in enabling participants to manage fluctuating medical needs without withdrawing from work. As P8 explained:

*Flexibility… if it allows that a person can work from home and he's a wheelchair user, it will make him more productive because his environment at home will always be set up in a more comfortable environment than what work would be*.

Participants described managing ongoing medical complications, including bladder and bowel dysfunction and the prevention of pressure sores, which required structured routines and periodic adjustment during the workday. In such contexts, flexibility, was not a preference but a practical necessity. P1 reflected:

*Some days like maybe your bowels wouldn't have gone well or you've got an issue with your bladder and I think that's some sort of an accommodation needs to be made from that where you should be allowed to work from home if needed*.

Similarly, in a period of acute illness, P4 noted:

*I've been working from home without…without being absent…I had to empty my bladder every hour and that was not going to work in my office*.

Across cases flexible arrangements enabled participants to maintain professional contribution, while managing complex and unpredictable health demands.

#### 3.1.4 Wheelchairs and assistive devices

Assistive devices, particularly high-quality wheelchairs, were central to participant's account of mobility and workplace participation. P4 described their wheelchair as the “*greatest assistive device,”* highlighting its importance in everyday functioning.

Participants emphasized that not all wheelchairs were equivalent. P6 explained:

*The normal person sees a wheelchair as a wheelchair…But they don't understand it. The reason why you've got this expensive wheelchair, what it can do, and what impact that has on your life*.

Appropriate equipment was associated with comfort in professional settings. P8 noted that, “*if you can get there [to work] with equipment that's working, you feel ‘more confident'*.”

Despite this importance, high-quality wheelchairs were described as financially burdensome, with medical aid schemes covering only part of the cost. Participants without access to suitable equipment reported greater difficulty managing daily routines and workplace demands.

#### 3.1.5 Support networks

Participants described peer support groups as particularly valuable in the early stages following injury. Organizations such as the QuadPara Association of South Africa were mentioned for providing practical guidance and connection to others with similar experiences. P4′s reflected on the uncertainty of the post-rehabilitation period:

*I remember when I got out of the rehab I didn't know what I was going to do. I didn't know what kind of job can I do. I was really lost…I thought. It's going to be bad I'm going to live on SASSA [social grant]*.

Over time, participants described less reliance on formal peer-support groups and greater emphasis on support from family, partners, and close friends. Pl reflected:

*I think maybe just because at this stage of my journey, I'm not feeling like it's so necessary for me to specifically go out and make friends with people in wheelchairs… I mean, I do the run every year…It's always nice seeing other people there, but I don't think it's as important…as it was at the beginning of my journey*.

Across cases, sources of support shifted as participants moved through different stages of adjustment and employment.

#### 3.1.6 Inclusive policies

Participants emphasized the importance of inclusive workplace policies and participation in workplace decision-making processes. These policies included flexible working arrangements, access to adaptive equipment, and the design of accessible office spaces.

In discussing how workplace environments could be made more inclusive, participants 6, 8, and 9 referred to the concept of universal design. P8 described this as: “*the total idea of universal accessibility and [design] fit for everybody”* noting that features such as ramps and adjustable desks enabled inclusion without segregating or excluding non-disabled employees. Universal design was thus framed as a practical approach to inclusion that benefitted a wide range of workers while addressing the specific needs of individuals with SCI.

Inclusivity was also understood in terms of representation and voice within organizational structures. Participant 1, 7, and 9 expressed a desire for greater representation of people with disabilities in leadership and decision-making roles. As P1 explained, “*I think having a manager who has the same disability as you makes a huge difference. Or at least not the same disability, just a disability, I think, makes a world of a difference to feeling understood and feeling that you can speak out about your issues.”* Representation was thus experienced as enabling both psychological safety and practical influence over workplace practices.

The absence of consultation with individuals with lived experience was highlighted through examples of poorly implemented adaptations. P4 described returning to work to find a newly constructed ramp that was unusable: “*They built a ramp… for me in my absence. When I got there, the ramp was steep. Like very steep, and I can't use this ramp.”* This example illustrated how well-intentioned but unilateral decisions could inadvertently reinforce exclusion when adaptations were not co-designed with those who would use them.

Participants also reflected on how individuals with SCI contributed to workplace environments beyond formal inclusion targets. Several participants described people with SCI as bringing heightened adaptability, problem-solving capacity, and perseverance developed through navigating daily constraints. As P1 noted:

*People that I've met, and maybe I've just seen one side of it, but all the people I've met in wheelchairs are the most resourceful people. They are the most, what would you call it? Resilient people?*

These reflections highlighted how participants understood their lived experience of SCI as a source of practical knowledge and perspective shaping how they approached work.

### 3.2 Social support and relational resilience

Participants consistently described relational support as central to sustaining alignment between health demands, workplace participation, and everyday functioning. Social and emotional support operated not merely as supplementary assistance, but as an ongoing mechanism through which workforce participation was stabilized during periods of structural or health-related strain.

#### 3.2.1 Managerial empathy and relational understanding

Workplace support emerged as a critical factor in successful workforce reintegration, with managers and colleagues playing a key role in shaping inclusive and responsive work environments. Three participants (P1, P3, P8) described working under managers who either had a disability themselves or had close family members with disabilities, which they believed contributed to these managers' heightened understanding and empathy. P3 explained:

*I‘ve been fortunate in that the people that I've worked for have had some exposure to people [with disabilities] like my current manager he's got a brother-in-law who's a quadriplegic who had a car accident and so he knows the whole story and he can empathise… and I think that is why I‘ve been in the team that I've been for so long*.

This exposure to disability appeared to enhance managerial awareness of everyday challenges, contributing to more responsive and accommodating workplace practices. This finding highlights the role of visibility and lived experience in shaping relational forms of inclusion within organizations.

#### 3.2.2 Family and social support network

Family and social support emerged as enduring facilitators of self-management and workforce reintegration for individuals with SCI. Across interviews, participants consistently identified family members and close friends as essential resources for both daily health management and long-term adjustment. P8 reflected on the role of family support in shaping post-injury trajectories:

*I think that you take two guys coming out that have had injuries about the same time and everything else and you see one's fantastic and another one's not. I am willing to guarantee you that the one that's doing well has 100% family support and friend support and the other guy is feeling sorry because he's not getting their support*.

These accounts highlight how sustained emotional and practical support from family and close social networks provided a foundation for managing health demands, maintain motivation, and remaining engaged in work. Relational resilience therefore emerged not as an individual trait, but as a capacity through ongoing interpersonal support and shared adaptation.

### 3.3 Agency, adaptation, and career growth

The final theme captures how participants enacted adaptive agency in response to fluctuating workplace, relational, and health demands. Rather than reflecting individual resilience in isolation, participants' agency emerged through ongoing negotiation, learning, disclosure, and adaptation within environments that variably enabled or constrained participation.

Participants consistently described acceptance of their injury as a critical turning point in both psychological adjustment and professional reintegration. Acceptance was not framed as resignation, but as an enabling shift that allowed participants to plan, set goals and reimagine their futures. As P8 explained,

*Everybody has to go through that process to get to acceptance of being in a chair, having a major life-changing situation, that does change your life. If they don't go through that and get to the point of acceptance, then obviously there's… that's when the complications start coming through, especially on the mental side*.

Across accounts, acceptance functioned as a foundation for rebuilding confidence, developing adaptive strategies, and re-engaging with professional life.

#### 3.3.1 Self-efficacy and adaptation

Participants described developing new forms of self-efficacy as essential to navigating everyday life following spinal cord injury. Rather than emerging as an inherent personal trait, confidence and independence were gradually cultivated through experience, problem-solving, and interaction with supportive environments. Growing independence was frequently described as central to effective self-management and reintegration into work and daily life. As P9 explained, “*I think that that independence is critical… that's probably the most important part around the self-management for me.”* These accounts suggest that self-efficacy developed through adaptive learning processes that enabled participants to manage health demands while sustaining engagement in work and everyday activities.

Functional limitations associated with SCI also required participants to seek assistance from others, involving a deliberate engagement with vulnerability in both personal and professional contexts. As P9 noted, “*You learn to adapt and you learn how to ask for help and you know, use the help where it's where it's offered.”* Similarly, P1 reflected, “*I've found in the workplace that if I don't just ask someone to help me… then I'm missing out on opportunities.”* In this sense, vulnerability functioned not as a loss of independence, but as a relational strategy that enabled collaboration, strengthened trust, and sustained workplace participation.

Establishing consistent routines was also described as central to participants' capacity for self-management. Daily routines provided stability in managing health needs while maintaining productivity. As P8 observed, “*That there's no other way, you know. And because as soon as you put something in routine, it becomes a habit.”* Although routines were individually enacted, participants emphasized that they were developed in response to environmental demands, workplace expectations, and relational support systems.

Taken together, self-efficacy, vulnerability, and routine functioned as interdependent components of adaptive agency rather than isolated personal attributes. Participants' capacity to remain engaged in work was therefore shaped not only by internal motivation, but also by relational support and environmental conditions that enabled confidence, sustainable routines, and collaborative problem-solving over time.

#### 3.3.2 Confidence building and career progression

Beyond acceptance, confidence emerged as foundational to career re-entry, progression, and professional self-advocacy. Participants described confidence as enabling them to articulate accommodations needs, pursue new career directions, and assert professional competence within workplace settings. Participants 1, 8, and 9 emphasized that confidence building was central to seeking employment, advancing careers, and establishing credibility within professional environments. P8 highlighted the importance of clarity, intention, and professional self-belief:

*I think that's my biggest one [piece of advice] is have the right attitude. Don't go in there with disability in the back of your mind. You go in there confident and make sure that you know what you want*.

This confidence also shaped participants' everyday work experiences, while simultaneously educating colleagues and managers about the practical realities of SCI and fostering more inclusive workplace cultures.

#### 3.3.3 Education and skills development

Education and skills development emerged as central strategies through which participants sustained career trajectories and adapted to changed physical and professional realities. P4 emphasized the importance of access to skills development, aligned with labor market demands:

*Spaces where you can equip people with a real world problem solving skills. The skills that are necessary on the job market. The skills that are in high demand on the market are necessary for people living with disabilities, because if you don't have skills, then it's very difficult to employ you*.

Participants across the sample highlighted the value of targeted training programs, workshops, and career-oriented resources that addressed both technical and soft skills. As P1 explained:

*Like you have a thousand things going on [post injury] and I think it would be such a valuable thing just to like help people figure out what are they going to do next, put together a CV and just having someone to come and you know…for morale, you know, come and talk and say it's not that scary going back into the workforce, that [it] takes a bit of planning [and] here are some tips and considerations*.

The importance of vocational support was further echoed by P4 when discussing what helps individuals with SCI return to work: “*…having the resources to equip yourself with skills suitable for where you want to work and where you are able to work*.” Such resources supported participants in preparing for labor market participation and navigating professional expectations with greater confidence and clarity.

#### 3.3.4 Workplace education and awareness

Several participants identified workplace disability education as a key mechanism for strengthening inclusion and sustaining career participation. As P9 explained:

*I think if you can build on overall awareness and then obviously specific awareness within a workspace or within certain environments, then again it helps to explain to people why certain things need to be a particular way and you know, once they understand, then they generally accept and move on*.

Workplace education fostered mutual understanding by clarifying accommodation needs and normalizing disability-related practices within teams. Across accounts, adaptation and growth were framed not as responses to limitation, but as active expressions of agency. Participants demonstrated how acceptance, confidence, continuous learning, and advocacy interacted to enable both individual career progression and incremental transformation of workplace environments. Importantly, participants' experiences demonstrated that structural conditions, relational supports, and adaptive agency rarely operated independently. Workforce sustainability emerged through the continual negotiation and recalibration of these domains, with disruption or misalignment in one domain frequently destabilizing participation across others. Reintegration therefore, appeared less as a stable outcome and more as an ongoing process of adaptive coordination.

### 3.4 Integrated findings

Taken together, these themes suggest that workforce reintegration cannot be explained through structural provision, relational support, or individual adaptation alone. Rather, sustained participation appears to depend on how these domains interact and are recalibrated over time. Building on this insight, the Adaptive Occupational Alignment model is introduced next.

#### 3.4.1 Adaptive occupational alignment: an integrative processual model

**Figure 1:**
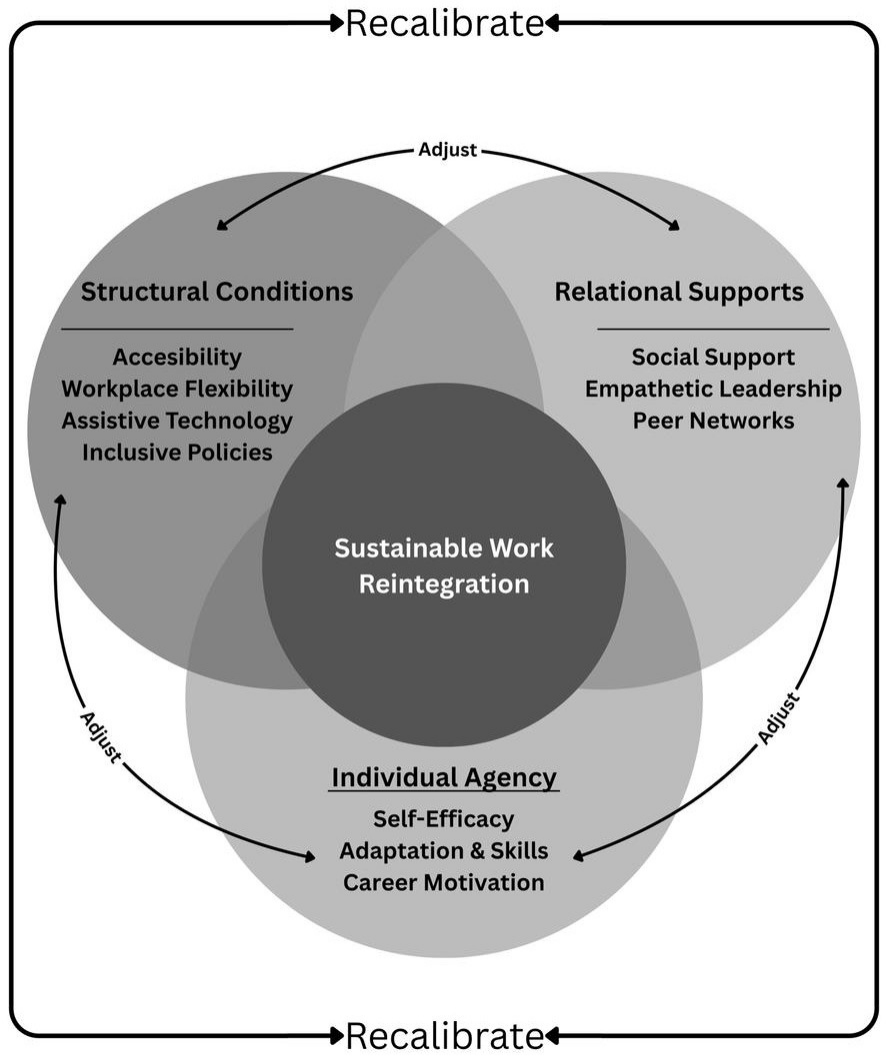
Adaptive occupational alignment model. The figure illustrates how sustained workforce participation emerges through the dynamic interaction of structural conditions, relational supports, and individual agency. Bidirectional influences reflect the adaptive recalibration required as health conditions, workplace practices, and social supports evolve over time.

Synthesizing the findings across the structural, relational and agency themes, this study advances a conceptual model termed Adaptive Occupational Alignment. The model (Figure 1) explains workforce reintegration following spinal cord injury as a multi-level, non-linear process through which sustained participation emerges from the dynamic alignment of structural conditions, relational supports, and individual agency.

Rather than conceptualizing return to work as a discrete transition from rehabilitation to employment, Adaptive Occupational Alignment positions reintegration as an ongoing process of recalibration. Participants' ability to remain engaged in work depended on how effectively health needs, workplace practices, transport access, assistive technologies, and social relationships were aligned in everyday practice. This alignment was neither automatic nor stable; it required continual negotiation in response to fluctuating health, organizational discretion, and uneven access to resources.

At the structural level, alignment was shaped by material and organizational conditions, including accessibility, transport, assistive devices, workplace flexibility, and policy implementation. These conditions were foundational but insufficient in isolation. Structural supports enabled sustained participation only when they remained responsive to changing health demands. For example, flexible and remote work arrangements allowed participants to manage medical complications without disengaging from employment, illustrating that structural alignment must accommodate fluctuation rather than assume stability.

At the relational level, alignment was mediated through interpersonal understanding and social support. Supportive managers, empathetic colleagues, peer networks, and family members functioned as stabilizing resources, particularly during periods of structural or health-related strain. Managerial empathy, often informed by personal exposure to disability, enabled discretionary flexibility and informal problem-solving beyond formal policy provisions. Over time, support structures evolved, with early reliance on peer networks transitioning toward sustained familial and workplace-based support, demonstrating the relationally embedded nature of occupational sustainability.

At the individual level, alignment was sustained through adaptive agency. Participants described developing acceptance, routines, self-efficacy, and career-oriented strategies that enabled them to manage health demands while maintaining professional identity. Importantly, agency was not framed as self-reliance in isolation but as the capacity to navigate vulnerability, disclose needs, and mobilize available supports. Confidence and skill development enabled participants to advocate for accommodations and pursue career growth, yet these capacities were shaped and reinforced by structural and relational conditions.

Crucially, the model highlights that misalignment in any one domain can destabilize workforce participation, even when other domains appear supportive. Strong motivation and social backing could not compensate for inaccessible transport or inadequate assistive equipment, while progressive policies failed in the absence of relational responsiveness. Conversely, alignment could be restored through adaptive recalibration, such as renegotiating work arrangements or strengthening support networks, illustrating that reintegration is an ongoing organizational accomplishment rather than a fixed achievement. In this way, Adaptive Occupational Alignment shifts attention from individual return-to-work readiness toward the relational and structural coordination required to sustain participation over time.

By foregrounding alignment as adaptive and relational, the model reframes workforce reintegration as a dynamic accomplishment sustained through continual recalibration across structural, relational, and individual domains rather than linear transitions from rehabilitation to employment.

## 4 Discussion

This study advances understanding of workforce reintegration following spinal cord injury by developing the conceptual model of Adaptive Occupational Alignment. While the findings affirm insights from the social model of disability (62), self-efficacy, and resilience theory, they also demonstrate that sustainable workforce participation is best understood as an ongoing, multi-level alignment process rather than as the product of structural reform, psychological strength, or adaptive endurance alone.

### 4.1 Structural conditions: beyond policy compliance toward adaptive alignment

Consistent with prior research (9, 13, 68), the findings confirm that structural conditions, both physical and institutional, remain decisive in shaping employment opportunities for people with SCI. Despite progressive legal frameworks in South Africa, participants continued to encounter inaccessible transport, poorly designed infrastructure, financial burdens associated with assistive devices, and uneven implementation of accommodation policies. These findings reinforce the social model of disability (48, 49), which situates disability within socially constructed barriers rather than individual impairment.

However, Adaptive Occupational Alignment extends the social model by demonstrating that structural provisions alone do not guarantee sustained inclusion. The findings show that even well-intentioned adaptations, such as ramps constructed without consultation or seating arrangements that inadvertently isolate employees, can undermine participation when they are not contextually and relationally aligned. Inclusion, therefore, cannot be reduced to compliance or infrastructure; it requires continuous adjustment to evolving bodily needs, organization routines, and interpersonal dynamics.

Workplace flexibility, particularly remote work, emerged as a critical mechanism for maintaining alignment. While previous literature has emphasized flexibility as a logistical accommodation (39, 60), this study illustrates how flexible arrangements function as adaptive structures that allow individuals to regulate health demands without disengaging from professional identity. Structural alignment, therefore, is not static accessibility but ongoing responsiveness.

### 4.2 Relational supports: reframing resilience as co-regulated

The findings further highlight the relational scaffolding that underpins sustained workforce participation. Family networks, peer groups, and empathetic managers played pivotal roles in stabilizing work engagement, particularly during periods of health fluctuation or structural strain. These insights align with research emphasizing relational dimensions of self-management (28, 72) and support resilience theory's understanding of adaptation as resource dependent (52).

Moreover, Adaptive Occupational Alignment reframes resilience as relationally enabled rather than individually possessed. Participants' capacity to persist at work was strengthened through managerial discretion, emotional support, and evolving social networks. Notably, support groups were most critical in early post-injury adjustment, while long-term participation relied increasingly on embedded familial and partner-based relationships. This temporal dimension complicates existing resilience accounts by showing that resilience shifts in form and source over time.

Our novel contribution here is the reframing of vulnerability. Participants who openly requested assistance and negotiated accommodations described more stable reintegration trajectories (27, 42). Rather than undermining independence, vulnerability functioned as a mechanism of alignment, facilitating trust, collaboration, and adaptive adjustment. This relational dynamic challenges dominant narratives equating independence with success and positions relational interdependence as foundational to occupational sustainability.

### 4.3 Agency and self-efficacy: contextually mediated capacity

The intrapersonal dimension of reintegration aligns with self-efficacy theory (50), as participants described confidence, acceptance, routine-building, and skill as critical to sustained employment. Consistent with Hammel et al. (51), participants who believed in their capacity to adapt were more likely to navigate workplace demands successfully.

Furthermore, the findings complicate purely psychological interpretations of self-efficacy. Confidence did not emerge in isolation; it was shaped by access to assistive technology, inclusive managerial practices, and supportive relationships. Adaptive Occupational Alignment, therefore, reconceptualises self-efficacy as a contextually mediated capacity, strengthened when structural and relational conditions enable agency, and weakened when misalignment persists.

Acceptance also emerged as a key psychological milestone. Participants described acceptance not as resignation but as an active cognitive shift enabling forward planning, advocacy, and career development. This cognitive shift suggests a processual sequence in which acceptance facilitates self-efficacy, which in turn supports adaptive agency. Such sequencing adds nuance to literature that often treats resilience and self-efficacy as parallel constructs rather than dynamically linked processes.

Furthermore, this study advances existing scholarship on career development post SCI by illustrating how individuals actively pursued retraining, workshops, and professional growth opportunities. These efforts were not merely compensatory but transformative, reframing participants as contributors and assets within their organizations. This pattern of career re-engagement challenges deficit-based discourses and aligns with calls to recognize the organizational value of disability inclusion (61).

### 4.4 Theoretical advancement: from domains to dynamic alignment

While previous scholarship has examined structural barriers, relational supports, and individual agency as distinct factors, this study demonstrates that sustainable workforce participation depends on their continuous interaction. Adaptive Occupational Alignment provides a process-oriented explanation of how these domains converge to sustain employment under conditions of health fluctuation and institutional constraint. Rather than conceptualizing reintegration as a transition completed at the point of return to work, the model positions it as an ongoing organizational and relational accomplishment. Misalignment in any domain can destabilize participation, even when other supports are present. Conversely, adaptive recalibration across domains can restore sustainability.

While frameworks such as Occupational Adaptation Theory and the Person-Environment-Occupation-Performance (PEOP) model have significantly advanced understanding of occupational participation and environmental fit, Adaptive Occupational Alignment differs in several important respects. First, the model conceptualizes workforce reintegration as an ongoing and relationally negotiated process rather than a discrete return-to-work outcome. Second, it foregrounds instability and recalibration, recognizing that participation remains vulnerable to disruption under fluctuating health and organizational conditions. Third, the model emphasizes the interaction between structural, relational, and agentic domains, demonstrating that sustainable workforce participation depends not on occupational fit alone, but on the continual coordination of these domains over time. In this way, Adaptive Occupational Alignment extends existing occupational frameworks by shifting attention from occupational fit to the ongoing maintenance of participation through dynamic alignment across multiple domains.

By foregrounding alignment as dynamic and relational, this model advances disability and occupational health scholarship beyond static inclusion frameworks. It offers a conceptual lens through which workforce participation is understood not as an individual achievement or policy outcome, but as a negotiated, multi-level process sustained through continual adjustment.

Building on these findings, workforce reintegration can be reframed as Adaptive Occupational Alignment: a dynamic process through which structural conditions, relational supports, and individual agency are continually recalibrated to sustain participation over time. Rather than treating return to work as a discrete outcome of rehabilitation or policy compliance, this study conceptualizes reintegration as an ongoing organizational and relational accomplishment.

### 4.5 Implications for occupational health and workplace practice

From an occupational health perspective, the findings suggest that sustainable workforce participation for individuals with SCI requires attention to alignment across structural, relational, and individual domains.

At the structural level, policies and accessibility measures must be adaptable to fluctuating health needs rather than designed as static compliance mechanisms. Flexible work arrangements, particularly hybrid and remote options, may function not only as accommodations but as preventive occupational health strategies that reduce secondary complications and preserve productivity.

At the relational level, managerial training and disability awareness initiatives appear to strengthen alignment by reducing misinterpretation of accommodation needs and fostering trust. The findings indicate that relational understanding may be as critical as physical infrastructure in sustaining inclusion.

At the individual level, vocational support services that incorporate psychological adjustment, skills development, and structured career guidance may enhance adaptive capacity. Importantly, these supports are most effective when embedded within enabling organizational environments rather than framed as compensatory interventions.

The findings further suggest that workforce reintegration interventions should move beyond one-time return-to-work planning toward longitudinal models of adaptive occupational coordination. Rehabilitation and occupational health services may benefit from incorporating periodic workplace reassessment, structured employer engagement, flexible work design, and integrated vocational support capable of responding to fluctuating health demands over time. In this sense, Adaptive Occupational Alignment offers a framework not only for understanding reintegration, but for informing the design of sustainable workplace and rehabilitation interventions.

### 4.6 Limitations and boundary conditions

Several limitations should be acknowledged. The sample comprised individuals who had successfully returned to work following SCI; therefore, the findings may not fully capture the experiences of those unable to re-enter the labor market. Future research could examine cases of persistent unemployment to further refine the model's explanatory boundaries. The study did not collect detailed clinical indicators such as American Spinal Injury Association (ASIA) Impairment Scale classifications, injury completeness, or injury etiology. Consequently, the influence of these clinical characteristics on workforce reintegration experiences could not be examined.

Additionally, the qualitative design and relatively small sample size limit generalizability. However, the study prioritizes conceptual development over statistical representation, offering a theoretically transferable model that may be tested quantitatively in future research.

Finally, the findings are situated within the South African policy and labor context. While Adaptive Occupational Alignment may be applicable to other settings and to other forms of chronic or episodic disability, cross-contextual validation would strengthen its generalizability.

### 4.7 Significance for disability and occupational health scholarship

By conceptualizing workforce reintegration as an ongoing process of adaptive alignment, this study moves disability and occupational health scholarship beyond binary notions of inclusion vs. exclusion. It provides a framework for understanding how work is continuously negotiated under conditions of bodily vulnerability, institutional constraint, and relational interdependence.

Adaptive Occupational Alignment thus offers both a theoretical lens and a practical orientation for scholars and practitioners seeking to design workplaces that sustain participation over time rather than merely facilitate initial return.

## 5 Conclusion

This study examined the lived experiences of individuals with spinal cord injury who successfully returned to the workforce, identifying how structural conditions, relational supports, and individual agency interact to sustain employment over time. While prior scholarship has examined these domains separately, the findings demonstrate that workforce reintegration is best understood as a dynamic process of Adaptive Occupational Alignment, in which continued participation depends on the ongoing alignment of health needs, organizational practices, and relational supports.

By reframing reintegration as an adaptive and relational process rather than a fixed post-rehabilitation milestone, this study advances disability and occupational health scholarship beyond static models of accommodation or individual resilience. The findings highlight that inclusion is not secured solely through policy compliance, physical accessibility, or personal determination, but through the everyday recalibration of workplace practices and support arrangements.

Although situated within the South African context, the Adaptive Occupational Alignment conceptual model developed here offers broader relevance for understanding employment sustainability among individuals living with chronic or episodic conditions. Future research may test and refine this Adaptive Occupational Alignment model across contexts and disability categories, further clarifying how workplaces can sustain participation under conditions of bodily vulnerability and organizational constraint.

## Data Availability

The datasets presented in this article are not readily available because due to the sensitive and personal nature of the narrative data and the ethical commitments made to participants regarding confidentiality and anonymity, the raw datasets are not publicly available. De-identified excerpts of the data are included within the article to illustrate key themes and support the analysis. Requests for access to anonymised data may be considered on a case-by-case basis and must comply with the ethical approval granted by the University's Research Ethics Committee: Social, Behavioural and Education Research (REC: SBER 31536), as well as participant confidentiality agreements. Requests to access the datasets should be directed to Chantell Ngobeni: chantellngobeni@sun.ac.za.
