# Supplementary material for: Adaptive occupational alignment: a processual model of workforce reintegration after spinal cord injury
Source: Front Public Health. 2026 Jul 16;14:1831601. doi: 10.3389/fpubh.2026.1831601 (PMC13422422; doi:10.3389/fpubh.2026.1831601)
Supplement: Supplementary file 1 [file Data_Sheet_1.pdf]

Appendix A. Interview Guide

Thank you for granting me the opportunity to interview you today. My primary interest is to understand the experiences and perspectives of individuals with spinal cord injury (SCI) who have successfully reintegrated into the workforce after rehabilitation. This interview will entail discussing your journey with self-care management practices and navigating employment post-rehabilitation. I aim to explore the challenges you've faced, the strategies you've employed, and your insights into fostering inclusive workplace environments for individuals with SCI. Your participation will contribute valuable insights to

improving support systems and policies for individuals with SCI in South Africa. Your responses will remain confidential, and you are encouraged to share openly. Are you comfortable proceeding with these topics?

1. How old are you now? How old were you at the time of your injury?
2. What is your level of spinal cord injury (e.g., cervical, thoracic, lumbar)?
3. What is your current employment status and job role?
4. Please describe your experience with self-care management since completing rehabilitation.
5. Please describe the strategies you have found most effective.
6. How has your spinal cord injury impacted your daily routine, including activities related to personal care and household tasks?
7. Could you share any challenges you've faced in maintaining employment since your injury? How have you navigated these challenges?
8. What support systems or resources have been most helpful to you in managing your health and work responsibilities?
9. How do you perceive your employer's understanding of your needs related to your spinal cord injury? Have workplace accommodations been sufficient?
10. Please describe a typical workday for you now compared to before your injury. What adjustments have you had to make?
11. What role do you believe self-management plays in your ability to maintain employment? Are there specific skills or strategies you've developed that you find particularly beneficial?
12. How do you balance the demands of work with your physical health needs? Are there specific routines or practices you follow to manage both effectively?
13. What factors do you believe contribute most to successful reintegration into the workforce after a spinal cord injury? Are there areas where you feel more support is needed?

14. How has your social support network influenced your journey back to work? Have relationships withfamily, friends, or colleagues played a role in your adjustment?
15. What advice would you give to other individuals with spinal cord injury who are navigating their return to work? Are there lessons learned or tips you would share based on your experience?
16. In your opinion, what improvements could be made in healthcare or workplace policies to better support individuals with spinal cord injury in achieving and maintaining employment?
