# Supplementary material for: Adaptive occupational alignment: a processual model of workforce reintegration after spinal cord injury
Source: Front Public Health. 2026 Jul 16;14:1831601. doi: 10.3389/fpubh.2026.1831601 (PMC13422422; doi:10.3389/fpubh.2026.1831601)
Supplement: Supplementary file 2 [file Data_Sheet_2.pdf]

COREQ Item	Description	Where Addressed in Manuscript
Domain 1: Research Team and Reflexivity		
1. Interviewer/facilitator	Researcher two was the primary data collector.	Section 2.2 Data Collection
2. Credentials	Author (A.B.) specialises in research on inclusion with a focus on people living with disabilities and chronic illness, advocating for workplace equity and centring participant voices. The second author (EvM) is a medical practitioner with experience working in public and private settings with spinal cord injuries and rehabilitation programmes.	Section 2.3 Reflexivity and Researcher Positioning
3. Occupation	Author one: Professor<br>Author two: Medical Practitioner (student)	Section 2.3 Reflexivity and Researcher Positioning
4. Gender	Author one: Male<br>Author two: Female	Section 2.3 Reflexivity and Researcher Positioning
5. Experience and training	Author one: Qualitative Researcher and published.<br>Author two: Student researcher	Section 2.3 Reflexivity and Researcher Positioning
6. Relationship established	Author Two has worked in rehabilitation and through her established networks, recruitment was initiated.	Section 2.1 Sampling and Participants
7. Participant knowledge of the interviewer	Author two was known to certain participants. Through previous professional engagements.<br>No knowledge of Author one.	Section 2.3 Reflexivity and Researcher Positioning
8. Interviewer characteristics	Author (EvM - female) is a medical practitioner and while this positioning provided contextual insight into participants' accounts it also introduced potential power asymmetries and interpretive bias.	Section 2.3 Reflexivity and Researcher Positioning
Domain 2: Study Design		

9. Methodological orientation and theory	The study was underpinned by an interpretivist qualitative orientation, drawing on a qualitative multiple case study design.	Section 2 Methodology
10. Sampling	A snowball sampling strategy was employed, initiated through author (EvM) professional networks.	Section 2.1 Sampling and Participants
11. Method of approach	Participants were recruited through email and telephone.	Section 2.1 Sampling and Participants
12. Sample size	10 participants were included (3 female, 7 male), aged between 21 and 60 years.	Section 2.1 Sampling and Participants
13. Non-participation	No dropouts were recorded or declines to participate were received.	
14. Setting of data collection	Interviews were conducted online and in person at the participants preference and choice.	Section 2.2 Data Collection
15. Presence of non-participants	Interviews were done in private with only the participant and interviewer present	
16. Description of sample	Injury levels were distributed across cervical (n=5) and thoracic (n=5) categories.	Section 2.1 Sampling and Participants injury level
17. Interview guide	Questions/prompts provided	Appendix A: Semi-Structured Interview Guide
18. Repeat interviews	No repeat interviews were conducted	
19. Audio/visual recording	Teams audio and visual recording function was used and where in person and audio recorder with Mp3 recoding abilities were used.	Section 2.2 Data Collection
20. Field notes	Field notes were taken to capture real time observations.	Section 2.2 Data Collection
21. Duration	Length of interviews were between 60-90 minutes	Section 2.2 Data Collection
22. Data saturation	Discussion of saturation	Section 2.4 Data Analysis
23. Transcripts returned	Interviews were transcribed and returned to participants for review	Section 2.2 Data Collection
Domain 3: Analysis and Findings		
24. Number of data coders	Author one and Author two coded the data	Section 2.4 Data Analysis

25. Description of coding tree	The coding framework was developed iteratively through thematic analysis, incorporating both inductive and deductive codes that were progressively organised into higher-order thematic categories.	Section 2.4 Data Analysis
26. Derivation of themes	Themes were refined collaboratively by both researchers to ensure coherence, internal consistency, and theoretical sensitivity.	Section 2.4 Data Analysis
27. Software	Analysis was conducted manually with data management through Atlas ti.	Section 2.4 Data Analysis (ATLAS.ti)
28. Participant checking	Participants were not consulted on the findings.	
29. Quotations presented	Quotations are used as part of the findings.	Section 3 Findings
30. Data and findings consistent	Themes were refined collaboratively by both researchers to ensure coherence, internal consistency, and theoretical sensitivity.	Section 3 Findings
31. Clarity of major themes	Major themes were clearly presented through three interrelated thematic domains: structural conditions, relational supports, and adaptive agency, each supported by participant quotations and linked analytically to the broader framework of Adaptive Occupational Alignment.	Section 3 Findings
32. Clarity of minor themes	Minor themes, variations in experience, and divergent trajectories were incorporated throughout the analysis to reflect the complexity and non-linearity of workforce reintegration following SCI, particularly where participants described differing forms of workplace support, disclosure, adaptation, and occupational sustainability.	Section 3 Findings and Discussion
